# Astaxanthin Attenuates Environmental Tobacco Smoke-Induced Cognitive Deficits: A Critical Role of p38 MAPK

**DOI:** 10.3390/md17010024

**Published:** 2019-01-03

**Authors:** Xia Yang, An-Lei Guo, Yi-Peng Pang, Xiao-Jing Cheng, Ting Xu, Xin-Rui Li, Jiao Liu, Yu-Yun Zhang, Yi Liu

**Affiliations:** Jiangsu Key Laboratory of New Drug Research and Clinical Pharmacy, Xuzhou Medical University, Xuzhou 221004, China; XiaYangco@163.com (X.Y.); gal303827537@163.com (A.-L.G.); xzmu_pyp@163.com (Y.-P.P.); 15771378196@163.com (X.-J.C.); xutingt@126.com (T.X.); 15266427365@163.com (X.-R.L.); 15298370510@163.com (J.L.); 15094359563@163.com (Y.-Y.Z.)

**Keywords:** astaxanthin, cigarette smoke exposure, p38 MAPK, antioxidant inflammatory, synaptic-associated plasticity

## Abstract

Increasing evidence indicates that environmental tobacco smoke (ETS) impairs cognitive function and induces oxidative stress in the brain. Recently, astaxanthin (ATX), a marine bioactive compound, has been reported to ameliorate cognitive deficits. However, the underlying pathogenesis remains unclear. In this study, ATX administration (40 mg/kg and 80 mg/kg, oral gavage) and cigarette smoking were carried out once a day for 10 weeks to investigate whether the p38 MAPK is involved in cognitive function in response to ATX treatment in the cortex and hippocampus of ETS mice. Results indicated that ATX administration improved spatial learning and memory of ETS mice (*p* < 0.05 or *p* < 0.01). Furthermore, exposure to ATX prevented the increases in the protein levels of the p38mitogen-activated protein kinase (p38 MAPK; *p* < 0.05 or *p* < 0.01) and nuclear factor-kappa B (NF-κB p65; *p* < 0.05 or *p* < 0.01), reversed the decreases in the mRNA and protein levels of synapsin I (SYN) and postsynaptic density protein 95 (PSD-95) (all *p* < 0.05 or *p* < 0.01). Moreover, ATX significantly down-regulated the increased levels of pro-inflammatory cytokines including interleukin-6 (IL-6) and tumor necrosis factor (TNF-α) (all *p* < 0.05 or *p* < 0.01). Meanwhile, the increased level of malondialdehyde (MDA) and the decreased activities of superoxide dismutase (SOD), glutathione (GSH), and catalase (CAT) were suppressed after exposure to ATX (all *p* < 0.05 or *p* < 0.01). Also, the results of the molecular docking study of ATX into the p38 MAPK binding site revealed that its mechanism was possibly similar to that of PH797804, a p38 MAPK inhibitor. Therefore, our results indicated that the ATX might be a critical agent in protecting the brain against neuroinflammation, synaptic plasticity impairment, and oxidative stress in the cortex and hippocampus of ETS mice.

## 1. Introduction

Environmental tobacco smoke (ETS), the combination of the side-stream smoke emitted from the burning end of a tobacco product and the mainstream smoke exhaled by the smoker, contains more than 6000 chemicals that are harmful to human body and may lead to many serious health problems, such as cognitive impairment and dementia [1,2]. For instance, compared with nonsmokers, smokers are reported to have remarkably decreased prefrontal attention network activity, and such a deficit is related with the length of smoking time [3]. Moreover, pregnant women exposed to tobacco smoke may present fetal neurobehavioral damages [4].

Although the pathogenesis of cognitive impairments due to tobacco smoke exposure has not been completely understood, several factors have been implicated such as oxidative stress and inflammation. For instance, long-term exposure to tobacco smoke led to oxidative stress [5]. Oxidative stress, which is mainly attributable to excessive generation of reactive oxygen species (ROS), mediates the activation of the mitogen-activated protein kinases (MAPKs) MAPK signaling cascades, especially the p38 MAPK pathway. As an important member of the MAPK family, p38 MAPK has been demonstrated to play a key role in nuclear factor – kappa B (NF-Κb) activation and pro-inflammatory expression [6]. NF-κB, one of the ubiquitous transcriptional factors, is the main medium that leads to the enlargement of inflammatory responses and then promotes the expression of proinflammatory cytokines like TNF-α and IL-6 [7,8]. Furthermore, researches demonstrate that the tobacco smoking induced oxidative stress and inflammation are involved in brain dysfunction [9]. Meanwhile, excessive ROS and inflammatory cytokines can impair hippocampal structure and function on learning and memory-related synaptic plasticity and neurogenesis [10]. Therefore, we hypothesized that attenuation of oxidative stress and inflammation might reverse the cognitive impairment induced by ETS.

Astaxanthin (ATX), a naturally occurring red carotenoid pigment, is abundant in red yeast *Phaffia rhodozyma*, green algae *Haematococcus pluvialis* and many kinds of marine organisms such as salmon and lobsters [11,12]. ATX has hydrophobic polyunsaturated polar structure on both ends of the conjugated olefins structure that facilitate its precise positioning within cell membranes and circulating lipoproteins, before exhibiting potent antioxidant functions as a powerful scavenger of oxygen free radicals so as to decrease oxidative stress and lipid peroxidation [13,14]. Recent studies revealed that ATX can relieve ischemia-related injury in brain tissue by suppressing oxidative stress, glutamate release, and anti-apoptosis [15]. Furthermore, some researches find that ATX can exert neuroprotective effects by weakening neuroinflammation [16]. More excitingly, ATX can attenuate subarachnoid hemorrhage induced neuroinflammation in rats and improve hippocampal plasticity and cognitive functions in male C57BL/6J mice [17]. However, the protective effects of ATX against ETS-induced cognitive decline have not been investigated. Therefore, the current work was designed to evaluate whether ATX can alleviate ETS-induced cognitive decline, and investigate the mechanisms involved.

## 2. Results

### 2.1. Effects of ATX Treatment on Exposure to ETS Induced Cognitive Decline

**Figure 1:**
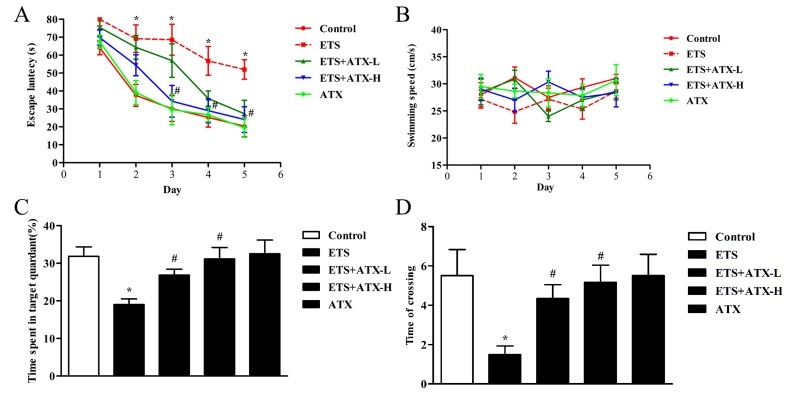
Effects of chronic astaxanthin (ATX) treatment on environment tobacco smoke (ETS) induced cognitive decline (*n* = 12). (**A**) Escape latency appeared during the training and the probe sessions. Data are reported as mean ± SE. (* *p* < 0.05) versus Group Control at the corresponding days; ^#^ *p* < 0.05 versus Group ETS at the corresponding days). (**B**) The swimming speed among the four groups during the five-day period. Data are reported as mean ± SE (*p* > 0.05). (**C**) The percentage of time spent in the target quadrant during the probe trial. Data are reported as mean ± SE. (* *p* < 0.05 versus Group Control; ^#^ *p* < 0.05 versus Group ETS). (**D**) The number of crossings of the platform area. Data are reported as mean ± SE (* *p* < 0.05 versus Group Control; ^#^ *p* < 0.05 versus Group ETS).

In order to investigate whether ATX could improve the cognitive impairments induced by ETS, we evaluated the learning and memory by the Morris water maze (MWM) test. The trained mice in all groups showed a decrease in mean escape latency during the learning trials (Figure 1A), and from the second day to the fourth day, an apparent elevation appeared in transfer latency in the ETS groups compared with the control group (all *p* < 0.05; Figure 1A). On the fifth day, the escape latency significantly elevated in ETS group compared with the control group *(p* < 0.05; Figure 1A). Meanwhile, ATX treatment (40 mg/kg and 80 mg/kg) significantly inhibited the elevation of escape latency in the ETS mice (all *p* < 0.05; Figure 1A). Administration with ATX (80 mg/kg) alone exhibited no visible difference in the escape latency compared with the control mice (*p* > 0.05; Figure 1A).

The probe trial was performed on the fifth day. In the ETS mice, the percentage of time spent in the target quadrant (*p* < 0.05; Figure 1C) and the number of crossings of the platform area (*p* < 0.05; Figure 1D) appeared to decrease in comparison with the control mice, while the decrease of the percentage of time spent in the target quadrant (*p* < 0.05; Figure 1C) and the number of crossings of the platform area (*p* < 0.05; Figure 1D) were prevented by ATX (40 mg/kg and 80 mg/kg) treatment. ATX (80 mg/kg) alone treated mice presented no visible differences in comparison with the control mice (*p* > 0.05; Figure 1C,D), suggesting that ATX itself had no influence on the learning and memory in the control group. The swimming speed exhibited similar performance among the five groups during the five-days MWM test (all *p* > 0.05, Figure 1B), which indicates that the differences in escape latency, the number of crossings, and the time spent in the target quadrant do not affect the movement defects.

### 2.2. Effects of ATX Treatment on Exposure to ETS Induced Parameters of Oxidative Stress in the Mouse Brain

**Figure 2:**
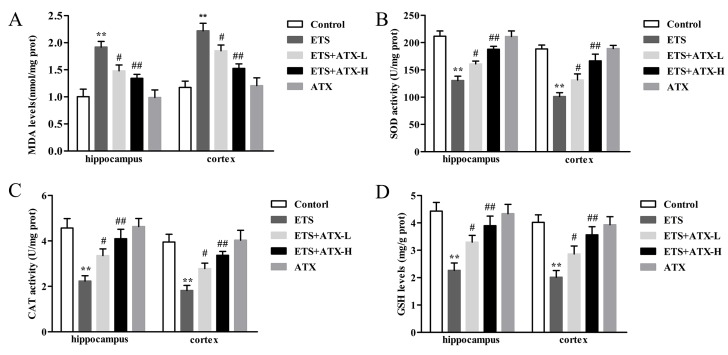
Effects of ATX on parameters of oxidative stress in the mouse brain (*n* = 12). (**A**) The level of MDA. Data are reported as mean ± SE. (** *p* < 0.01 versus Group Control; ^#^ *p* < 0.05 versus Group ETS; ^##^ *p* < 0.01 versus Group ETS). (**B**) The activity of SOD. Data are reported as mean ± SE. (** *p* < 0.01 versus Group Control; ^#^ *p* < 0.05 versus Group ETS; ^##^ *p* < 0.01 versus Group ETS). (**C**) The activity of CAT (** *p* < 0.01 versus Group Control; ^#^ *p* < 0.05 versus Group ETS; ^##^ *p* < 0.01 versus Group ETS). (**D**) The level of GSH. Data are reported as mean ± SE. (** *p* < 0.01 versus Group Control; ^#^ *p* < 0.05 versus Group ETS; ^##^ *p* < 0.01 versus Group ETS).

The MDA levels, SOD activities, CAT activities and GSH levels were detected to investigate whether ATX have the effects on the ETS exposed brain antioxidant system. According to the results described, the ETS mice presented a remarkable increase in the MDA levels (*p* < 0.01, Figure 2A) and a striking decrease in the SOD activities (*p* < 0.01, Figure 2B), CAT activities (*p* < 0.01, Figure 2C) and GSH levels (*p* < 0.01, Figure 2D) in the hippocampus and prefrontal cortex in comparison with the control group. Administration with ATX (40 mg/kg and 80 mg/kg) inhibited the ETS caused elevation of MDA levels (*p* < 0.05 or *p* < 0.01; Figure 2A) and prevented the ETS caused decrease of SOD activities (*p* < 0.05 or *p* < 0.01; Figure 2B), CAT activities (*p* < 0.05 or *p* < 0.01; Figure 2C) and GSH levels (*p* < 0.05 or *p* < 0.01; Figure 2D) in the hippocampus and prefrontal cortex. ATX (80 mg/kg) alone treatment presented no difference in these parameters of oxidative stress in comparison with the control mice in the hippocampus and prefrontal cortex (*p* > 0.05; Figure 2). These results indicated that chronic exposure to ETS caused oxidative stress in mice, and ATX treatment could attenuate the ETS caused oxidative stress.

### 2.3. Effects of ATX Treatment on Exposure to ETS-Induced Inflammation in the Hippocampus and Prefrontal Cortex

**Figure 3:**
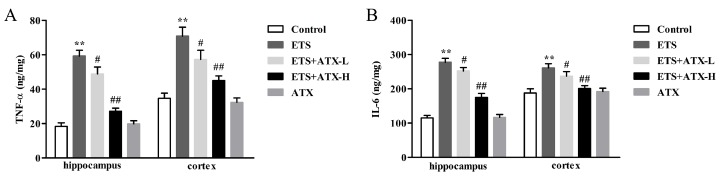
Effects of ATX on inflammation in the hippocampus and cortex (*n* = 12). (**A**) The levels of TNF-α. Data are reported as mean ± SE. (** *p* < 0.01 versus Group Control; ^#^ *p* < 0.05 versus Group ETS; ^##^ *p* < 0.01 versus Group ETS). (**B**) The levels of IL-6. Data are reported as mean ± SE. (** *p* < 0.01 versus Group Control; ^#^ *p* < 0.05 versus Group ETS; ^##^ *p* < 0.01 versus Group ETS).

Inflammatory response is closely linked to the pathogenesis of cognitive disorder, which damages hippocampal synaptic plasticity by increasing the levels of pro-inflammatory cytokines. Thus, the effects of ATX on ETS induced alteration of the levels of inflammatory factors (such as TNF-α and IL-6) in the brain were tested by ELISA. The levels of TNF-α (*p* < 0.01; Figure 3A) as well as IL-6 (*p* < 0.01; Figure 3B) were found to be increased remarkably in the hippocampus and cortex in ETS mice in comparison with control mice, while ATX (40 mg/kg and 80 mg/kg) administration attenuated the ETS induced increase in the levels of TNF-α (*p* < 0.05 or *p* < 0.01; Figure 3A) and IL-6 (*p* < 0.05 or *p* < 0.01; Figure 3B) in the hippocampus and cortex. ATX (80 mg/kg) treatment alone did not change inflammation levels in the hippocampus and cortex in comparison with the control group (*p* > 0.05; Figure 3A and B). These results inferred that ETS caused inflammatory response, and ATX could inhibit ETS caused inflammatory response.

### 2.4. Effects of ATX on the Expressions of NF-κB p65 in the Hippocampus and Prefrontal Cortex

**Figure 4:**
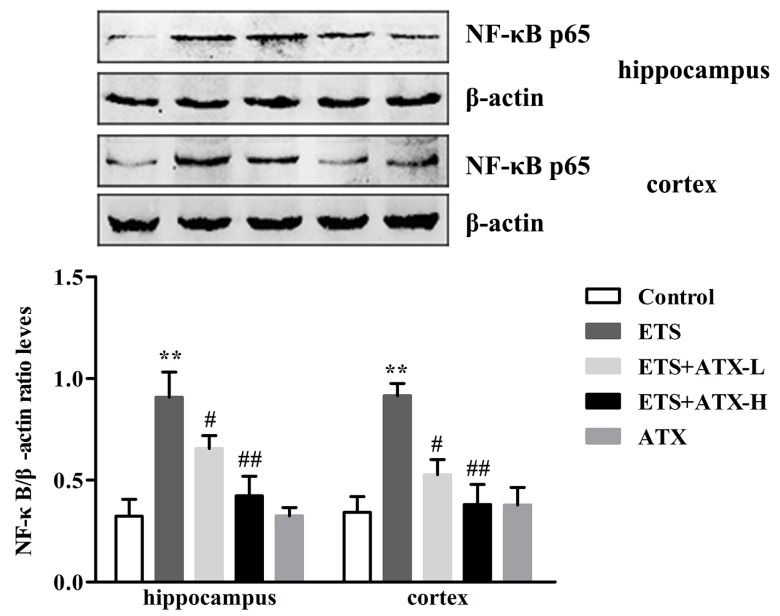
Effects of ATX on the expressions of NF-κB p65 in the cortex and hippocampus (*n* = 12). The levels of NF-κB p65 in the hippocampus and cortex of mice. Data are reported as mean ± SE. (** *p* < 0.01 versus Group Control; ^#^ *p* < 0.05 versus Group ETS; ^##^ *p* < 0.01 versus Group ETS).

NF-κB p65 expression was carried out to study the potential mechanisms of the neuroprotective changes in ATX treatment of ETS caused cognitive impairment. As shown in Figure 4, there was an obvious enhancement in NF-κB p65 levels (*p* < 0.01) in the hippocampus and cerebral cortex in ETS mice compared with control mice and this enhancement was repressed by ATX (40 mg/kg and 80 mg/kg) treatment (*p* < 0.05 or *p* < 0.01). Treatment with ATX (80 mg/kg) alone exhibited no difference compared to the control mice (*p* > 0.05). These results suggested that ETS enhanced the levels of the NF-kB, and ATX administration could prevent the enhancement of the levels of the NF-κB p65.

### 2.5. Effects of ATX on the Expressions of p38 MAPK and p- p38MAPK in the Hippocampus and Prefrontal Cortex of ETS Mice

**Figure 5:**
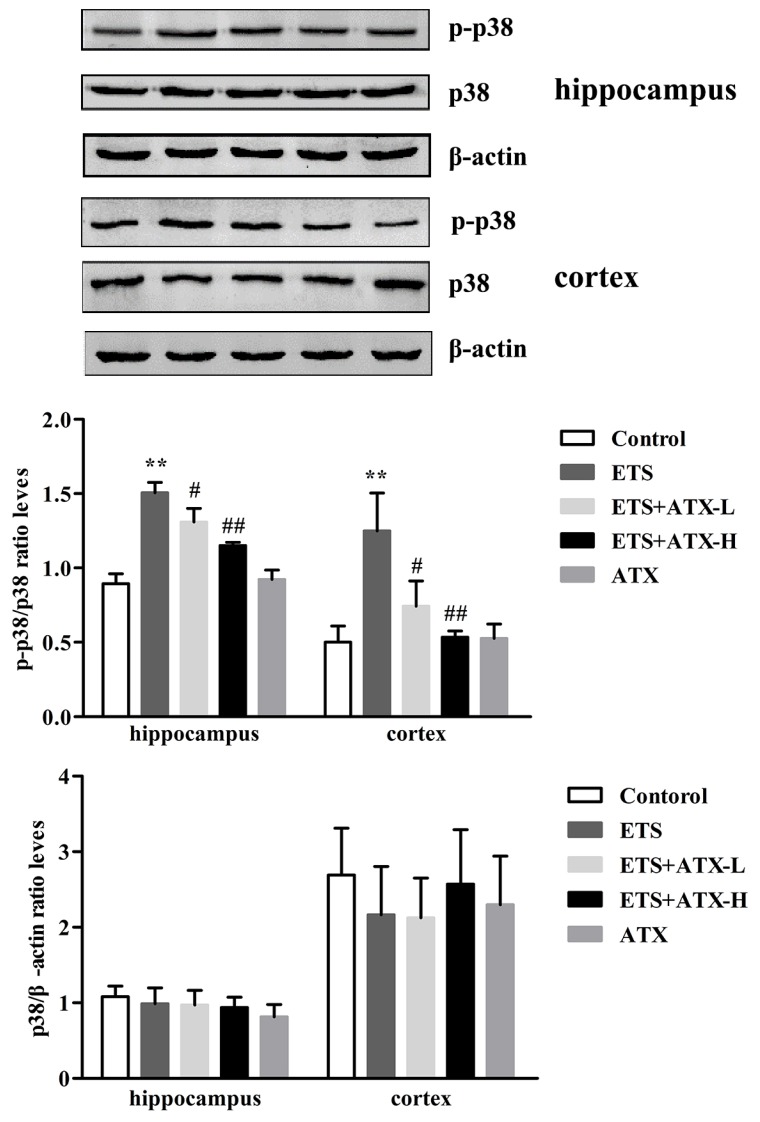
Effects of ATX on the expressions of p38 and p-p38 in the cortex and hippocampus (*n* = 12). The levels of p-p38 in the hippocampus and cortex of mice. Data are reported as mean ± SE. (** *p* < 0.01 versus Group Control; ^#^ *p* < 0.05 versus Group ETS; ^##^ *p* < 0.01 versus Group ETS).

The protein expression of total-p38 MAPK and p- p38 MAPK in the hippocampus and prefrontal cortex were tested by Western blot and the results are shown in Figure 5. In the ETS mice, the levels of phosphorylated p38 MAPK were remarkably increased in the hippocampus and cerebral cortex in comparison with the control mice (*p* < 0.01), while the increased levels of phosphorylated p38 MAPK were prevented in the ATX (40 mg/kg and 80 mg/kg) mice (*p* < 0.05 or *p* < 0.01). ATX (80 mg/kg) alone groups exhibited no obvious difference compared to the control group (*p* > 0.05). The levels of the total- p38 MAPK exerted no obvious differences among all groups in the hippocampus and cerebral cortex (*p* > 0.05). These results indicated that ETS caused the excessive activation of p38 MAPK, and ATX could inhibit the ETS caused the activation of p38 MAPK.

### 2.6. Effects of ATX on the Expression of SYN mRNA and PSD-95 mRNA in the Mouse Brain of ETS mice

**Figure 6:**
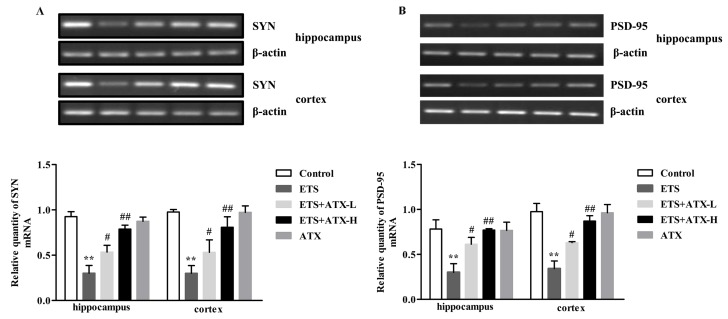
Effects of ATX on the expression of SYN mRNA and PSD-95 mRNA in the mouse brain (*n* = 12). (**A**) The levels of SYN mRNA. Data are reported as mean ± SE. (** *p* < 0.01 versus Group Control; ^#^ *p* < 0.05 versus Group ETS; ^##^ *p* < 0.01 versus Group ETS). (**B**) The levels of PSD-95 mRNA. Data are reported as mean ± SE. (** *p* < 0.01 versus Group Control; ^#^ *p* < 0.05 versus Group ETS; ^##^ *p* < 0.01 versus Group ETS).

SYN and PSD-95 are two major synaptic associated proteins that can directly or indirectly affect cognitive function [18]. Accordingly, reverse transcriptase-PCR (RT-PCR) was used to estimate the levels of SYN and PSD-95 mRNA. In the ETS mice, the expression of SYN mRNA (*p* < 0.01; Figure 6A) and PSD-95 mRNA (*p* < 0.01; Figure 6B) were markedly down-regulated in the hippocampus and cortex compared with the control mice, while this down-regulation of the SYN mRNA (*p* < 0.05 or *p* < 0.01; Figure 6A) and PSD-95 mRNA (*p* < 0.05 or *p* < 0.01; Figure 6B) levels were elevated by ATX (40 mg/kg and 80 mg/kg) administration. ATX (80 mg/kg) alone had no influence on the expression of SYN mRNA (*p* > 0.05; Figure 6A) and PSD-95 mRNA (*p* > 0.05; Figure 6B) in comparison with the control mice. These results inferred that ETS led to a reduction of the SYN mRNA and PSD-95 mRNA, and ATX could reverse this change.

### 2.7. Effects of ATX on the Expression of Synaptic Proteins in the Mouse Brain

**Figure 7:**
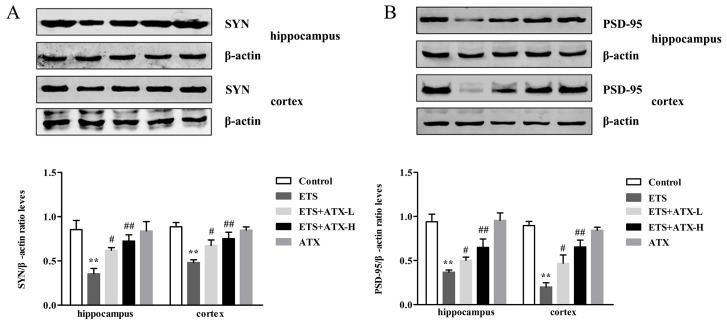
Effects of ATX on the expression of synaptic proteins in the mouse brain (*n* = 12). (**A**) The levels of SYN in the hippocampus and cortex of mice. Data are reported as mean ± SE. (** *p* < 0.01 versus Group Control; ^#^ *p* < 0.05 versus Group ETS; ^##^ *p* < 0.01 versus Group ETS). (**B**) The levels of PSD-95 in the hippocampus and cortex of mice. Data are reported as mean ± SE. (** *p* < 0.01 versus Group Control; ^#^ *p* < 0.05 versus Group ETS; ^##^ *p* < 0.01 versus Group ETS).

In order to detect whether ATX could protect synaptic plasticity from ETS impairment Western blot was used to examine the expression of SYN and PSD-95 proteins in the hippocampus and cortex. In the ETS mice, SYN (*p* < 0.01; Figure 7A) and PSD-95 (*p* < 0.01; Figure 7B) were noticeably reduced in the hippocampus and cortex compared with the control mice, while treatment with ATX (40 mg/kg and 80 mg/kg) inhibited this reduction of both SYP (*p* < 0.05 or *p* < 0.01) and PSD-95 (*p* < 0.05 or *p* < 0.01) expressions in ETS mice. Administration with ATX (80 mg/kg) alone presented no difference in SYP and PSD-95 expressions compared with the control mice (*p* > 0.05). These results inferred that ATX could prevent ETS induced changes of the SYN and PSD-95 protein expressions.

### 2.8. Effects of ATX on the Structure and Morphology of the Hippocampal Neurons

**Figure 8:**
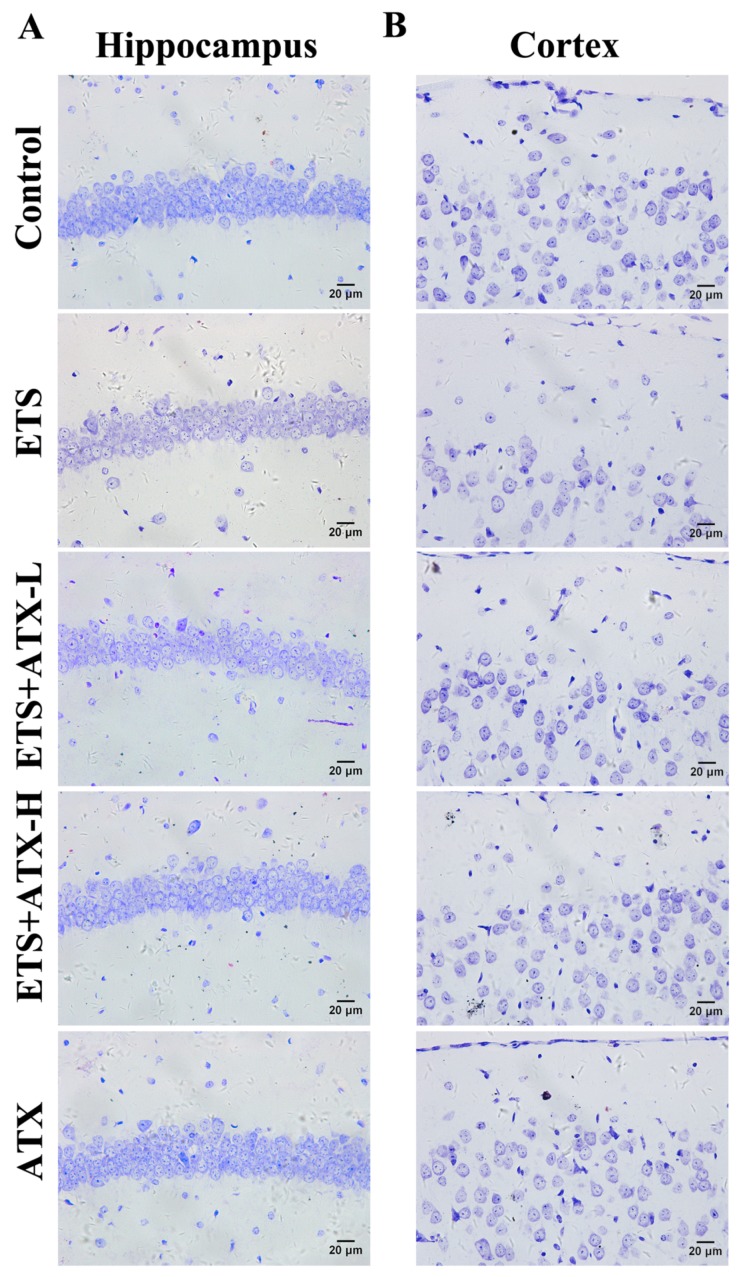
Effects of ATX on the structure and morphology of the hippocampal neurons (*n* = 12). Nissl stained neurons in the hippocampal CA1 subfield (**A**) and cortex (**B**). Bar = 20 μm.

Microphotographies of the cerebral cortex and the hippocampal CA1 subfield in each group are shown in Figure 8. In the ETS group, no obvious differences were observed in the neurons in the cerebral cortex in comparison with the control group. Meanwhile, no obvious differences were found in intact neuron counts in the cerebral cortex among the groups (Figure 8B). In contrast, in the hippocampal CA1 subfield of ETS mice, the neurons appeared in a noticeably wrinkled, irregular pattern, and a weak staining effect, and most Nissl bodies were lost, which inferred that extensively they were injured or dead (Figure 8A). Also, a significant decrease in the number of surviving neurons was observed in the ETS mice as compared to the control mice, while ATX (40 mg/kg and 80 mg/kg) treatment remarkably attenuated this decrease in ETS mice. Additionally, the ATX (80 mg/kg) group alone and the control group showed no difference in the number of surviving neurons, which revealed that ATX itself had no effect on the neurons in different areas of brain.

### 2.9. Molecular Docking Studies

**Figure 9:**
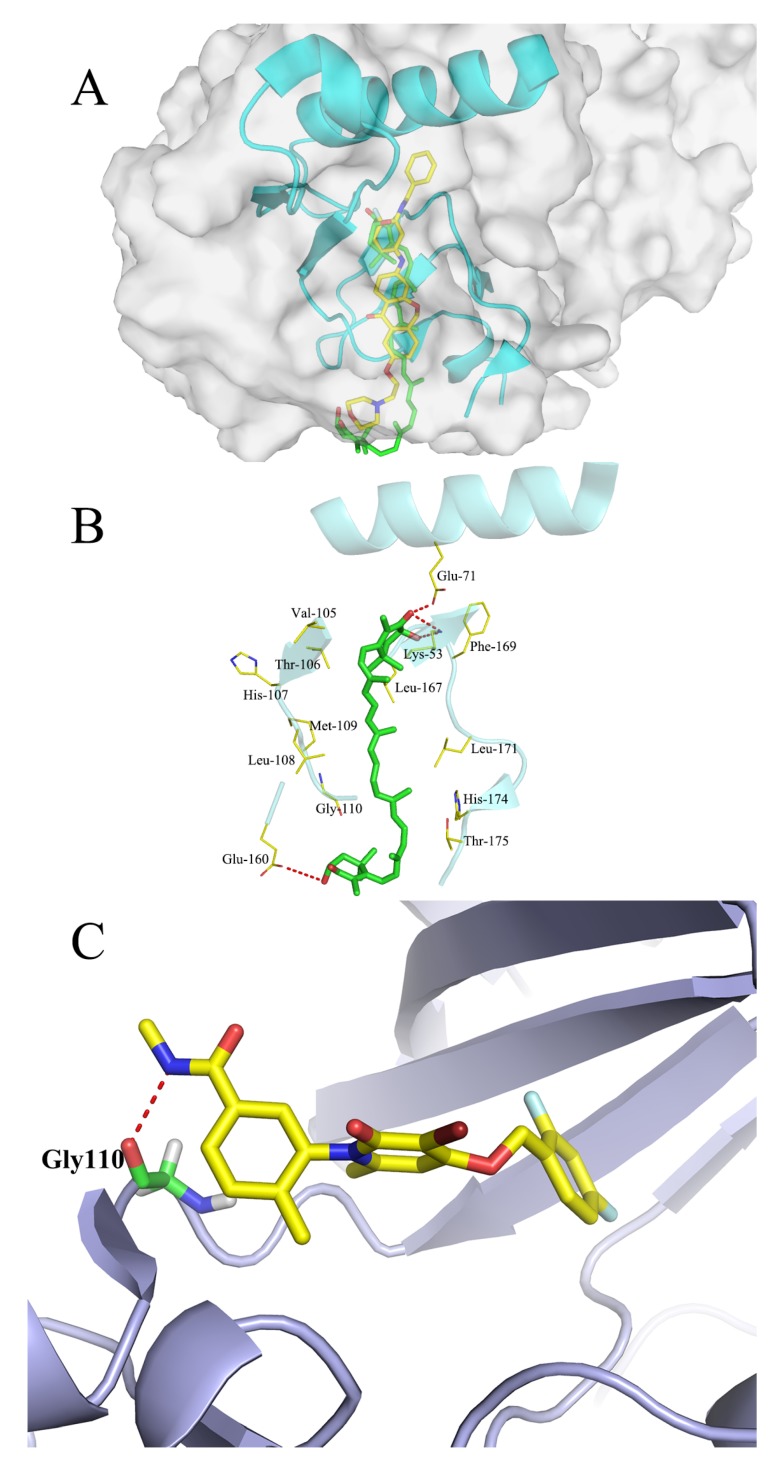
Molecular docking model for ATX (green and stick) with the active site of the ATP pocket of p38 alpha and the p38 alpha with the p38 inhibitor PH797804. (**A**) The whole picture of the molecular docking model for ATX with the p38 alpha. (**B**) Molecular docking model for ATX with the p38 alpha. Highlighting the hydrogen bonds (red dashed lines) coordination between the oxygen atoms in ATX and residues Glu-71 and Arg-49. (**C**) Molecular docking model for p38 MAPK with p38 MAPK inhibitor PH797804 binding pocket. The red color bonds indicate hydrogen bonds.

The interactions between ATX and human p38 and the interaction between human p38 alpha and p38 inhibitor PH797804 are shown as Figure 9. The p38 alpha and ATX docking pocket was formed by the residues of Glu-71, Leu-167, Phe-169, Leu-171, Thr-175, Arg-49, Leu-108, Met-109, Thr-106 and Leu-104, and a hydrogen bond was formed to Glu-71 from helix C. Meanwhile, another two hydrogen bonds were formed between the side chain of Arg-49 and the opposite end of ATX. p38 alpha and p38 inhibitor PH797804 docking pocking were formed by a hydrogen bond of Gly-110. The docking result demonstrated that ATX occupied the active site of the p38 and generated an interaction with surrounding amino acids like p38 inhibitor PH797804.

## 3. Discussion

According to the present research, chronic ATX administration reversed the ETS-induced cognitive deficits of mice. Notably, we found that ATX administration normalized the oxidative stress markers, decreased the levels of inflammatory cytokines, phospho-p38 MAPK, and NF-κB p65 proteins in the hippocampus and prefrontal cortex. In addition, the levels of SYN and PSD-95 were increased in the prefrontal cortex and hippocampus of ATX-treated mice. What is more important is that p38 MAPK may be the key factor in the reduction of cognitive deficits. 

Previous studies have reported that the capacity for learning and memory were impaired by cigarette smoke exposure [19,20]. However, the mechanism of ETS-induced cognitive impairment remains unclear. ROS are closely related with neuroinflammation and synaptic plasticity impairment [21]. Chronic cigarette smoke exposure induced an excessive ROS generation followed by the loss of the dynamic balance between ROS generation and elimination [16,22]. As a marine bioactive compound, ATX is reported to have antioxidant and anti-inflammation properties [16]. The present data indicated that ETS induced impairment in learning and memory function was improved by ATX treatment. To our knowledge, it is the first report that ETS-induced cognitive deficits can be improved by ATX treatment and the p38 MAPK may be the key factor in the reduction of cognitive deficits.

Generally, the MWM test is widely applied to measure the spatial learning of rodents [23]. Some researches demonstrate that the performance in the MWM test is usually related with both neurotransmitter systems and drug effects [24]. Several studies have confirmed that long term exposure to tobacco smoke could cause cognitive deficits [19,25]. Importantly, ATX can enhance cognitive function and attenuate depression-like behavior. So, we used MWM to observe the ETS-induced cognitive deficits and explore the therapeutic effect of ATX. The result of MWM indicated that the mice exposed to ETS showed enhanced escape latency and reduced time spent in the target quadrant (revealing an impairment of spatial learning and memory), which is consistent with published results [26]. Above all, long-term administration with low or high doses of ATX markedly reversed these behavioral changes, suggesting that ATX is the potential to protect ETS-induced cognition damage.

What is well recognized is that oxidative damage plays a crucial role in many brain dysfunction diseases [27]. Importantly, the brain is particularly vulnerable to oxidative stress because of a relatively high production rate of ROS without commensurate levels of antioxidative defense [28]. Tobacco smoke contains a large number of ROS which can permeate the blood brain barrier and mobilize the antioxidant defenses [29]. In the current research, we found an elevation of MDA, and a reduction of GSH, SOD, and CAT activities in the cerebral cortex and hippocampus of ETS mice, which is consistent with published results [30]. It has been proved that several flavonoids have strong antioxidant properties and improve memory and learning [31]. Moreover, treatment with ATX could decrease the MDA level and increase the SOD level in aging rats [32]. Our results showed that, the MDA level was suppressed, but GSH content, SOD, and CAT activity were raised when chronic administration with ATX in the hippocampus and prefrontal cortex of ETS mice. Consequently, these results support the hypothesis that ATX can inhibit the chronic ETS-induced pro-oxidant–antioxidant disequilibrium contributing to cognition improvement.

p38 MAPK as a stress-activated kinase, is sensitive to various exogenous and endogenous stimulations, and highly responded to oxidative stress and proinflammatory cytokines [33]. In addition, recent studies have found that the activation of the p38 MAPK signaling pathway is closely related with neuronal death or apoptosis, which may be the main reason of cognitive dysfunction [34]. In the current work, we found that the phosphorylation level of p38 MAPK was remarkably raised in the hippocampus and prefrontal cortex of ETS mice. And the chronic ATX administration attenuated the p38 MAPK phosphorylation level. Thus, we speculate that the cognition impairment of ETS mice may contribute to oxidative stress and the activation of p38 MAPK, where the activation of p38 MAPK may be more important.

It is well established that inflammation and oxidative stress are intricately interrelated. Oxidative stress is considered to be a crucial factor in regulating proinflammatory signaling pathways [35]. Long-term exposed to ETS induced oxidative stress and the activation of NF-κB followed by the release of the pro-inflammatory [36]. In addition, many studies confirm that the activation of NF-κB and the release of inflammation cytokines play a key part in the cognitive dysfunction that may explain cognitive decline [37]. In the current research, we also detected that the mice exposed to tobacco smoke showed up-regulated levels of NF-κB p65 and TNF-α and IL-6. However, chronic treatment with ATX remarkably suppresses the expression of NF-κB p65 and attenuates the excessive release of TNF-α and IL-6 [38].

The alterations of structural plasticity of dendrites and spines in the hippocampus and prefrontal cortex were found as a result of cognition deficits [39]. Research indicates that morphological alterations in the brain development of mice exposed to smoke may disrupt neural prediction [40]. Generally, synaptic plasticity is associated with the synapse related proteins, including presynaptic SYN and postsynaptic PSD-95 [41]. In our research, the reduction in the expression of synaptic proteins was observed in ETS-exposed mice, which may result in cognitive impairment. However, the reduction in both SYN and PSD-95 levels in ETS exposure mice was remarkably overturned, by chronic administration with ATX. Both SYN and PSD-95 were regulated by the inflammatory response caused by p38 MAPK and NF-κB p65. Therefore, these neurochemical findings imply that the neuroprotective response of ATX is attributable to reducing the phosphorylation level of p38 MAPK and relieving inflammatory responses. Thus, cognitive impairment in ETS-exposed mice can be improved by increasing the level of plastic-related proteins (SYN and PSD-95).

In conclusion, these findings manifest that ATX exerted protective effects on the cognition decline caused by ETS in mice. These improvements in the behaviors and neurochemicals implied that supplementation with ATX-enriched food may be an effective novel therapy and provide a hopeful mitigation to chronic ETS-induced cognition decline. Administration of ATX reduced oxidative stress and inflammatory responses, as well as enhanced the synapse-related proteins in the hippocampus and prefrontal cortex of ETS mice, and p38 MAPK plays an important role in the protection process. Therefore, our results provide ideals for further studies on the anti-inflammatory or antioxidant aspects of ATX and ATX derivatives in CNS related diseases in the future.

## 4. Materials and Methods 

### 4.1. Reagents

ATX (97% purity) was purchased from Xi’an Fengzu Biotechnology Co., Ltd (Shaanxi, China) and dissolved in olive oil (1 mL/kg) immediately before use. MDA, SOD, GSH, CAT, and BCA assay kits were obtained from Nanjing Jiancheng Biotechnology Co., Ltd (Nanjing, China). Antibodies against phospho-p38 MAPK (T^180^/Y^182^), p38 MAPK, SYN, PSD-95 and NF-κB p65 were from Cell Signaling Technology Inc., (Danvers, MA USA) and β-actin was from ZSGB-BIO, Beijing, China. All other reagents were from Sigma-Aldrich (St. Louis, MO, USA) unless otherwise indicated.

### 4.2. Animals

**Figure 10:**
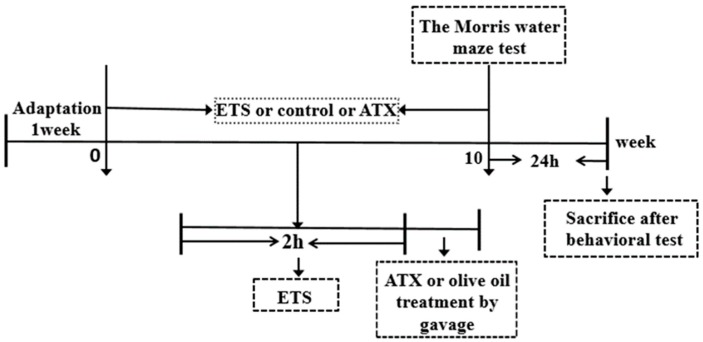
Schematic figure of the treatment protocol (*n* = 12).

Adult male Kunming mice weighing between 18 and 22 g were purchased from the Laboratory Animal Center, Xuzhou Medical University. The whole experimental schedule was depicted in Figure 10. The mice were housed with a 12 h light/dark cycle and free access to food and water under controlled temperatures 22 ± 2 °C and humidity 50 ± 10%. The animals were sacrificed within 24 h after the final test. All animal experiments in the current study were conducted in accordance with the Animal Ethics Committee, Xuzhou Medical University, China, and followed the National Institutes of Health Guidelines for the Care and Use of Laboratory Animals (Ethical approval number: XZMC2014-AN-39).

The mice were randomly divided into five groups according to their corresponding treatments (*n* = 12): (1) an ETS group: mice were exposed to ETS once a day for 2 h with an interval of 10 min between each cigarette, using 8 cigarettes per day for 10 consecutive weeks; (2) an ETS+ATX-L group: mice were exposed to ETS once a day for 2 h with an interval of 10 min between each cigarette, using 8 cigarettes followed by treatment with a low dose of ATX (40 mg/kg) once a day for 10 consecutive weeks; (3) an ETS+ATX-H group: mice were exposed to ETS once a day for 2 h with an interval of 10 min between each cigarette, using 8 cigarettes followed by treatment with a high dose of ATX (80 mg/kg) per day for 10 consecutive weeks; (4) an ATX group: mice were treated with 80 mg/kg ATX alone per day for 10 consecutive weeks; (5) a control group: under normal conditions with an equal volume of olive oil as ATX treatment once a day for 10 consecutive weeks. ATX was dissolved in olive oil before administration. Either ATX or the equal volume of olive oil was administered by oral gavage.

### 4.3. Smoke Generation

In the current study, smoke was generated according to previous descriptions [42]. Each cigarette contains 10 mg tar, 0.8 mg nicotine, and 10 mg carbon monoxide. After the mice were placed within a chamber (56.4 cm × 38.5 cm × 37.1 cm), four cigarettes (Jiangsu Tobacco Industrial Co. Ltd., China) were lit at one time and the chamber was shut down immediately, leaving a small hole (371 mm × 40 mm) in both ends for ventilation, and the cigarettes were burned up within 15 min. In order to keep an air flow inside the chamber, the smoke generated within the chamber was pumped by a noiseless extractor fan. The diluted side-stream smoke exposed to mice was adopted to imitate the ETS experienced for non-smokers.

### 4.4. Morris Water Maze (MWM)

The Morris water maze test was performed according to previous descriptions [43,44]. Mice were trained in a black circular pool (120 cm in diameter and 60 cm in height) filled with water (20–22 °C). The pool was divided into four quadrants with a clear 10 cm diameter escape platform hidden 1.5 cm beneath the surface in one of the quadrants. Training trials were conducted in the first consecutive four days, and the escape latency was recorded according to the time spent to reach the hidden platform. Then a probe trial was performed on the fifth day and the hidden platform was removed. The total time spent in each target quadrant was recorded.

### 4.5. Measurement of Oxidative Stress

After the behavioral assessments, the mice were sacrificed. The hippocampus and prefrontal cortex were dissected and homogenized (1:9 *w*/*v*) with cold normal saline (4 °C) to prepare 10% cerebral homogenate in an ice bath. The homogenized tissue was centrifuged at 4000 rpm at 4 °C for 10 min and the supernatant was collected for the following tests.

#### 4.5.1. Determination of Lipid Peroxidation

The MDA level was measured by supernatants reacted with thiobarbituric (TBA) to form thiobarbituric acid reactive substances using a commercial kit (Nanjing Jiancheng Bioengineering Institute, Nanjing, China) [45] and the absorption was determined at the wavelength of 532 nm.

#### 4.5.2. Determination of SOD Activity

The activity of SOD was assayed according to the method previously described [46]. Xanthine reacts with xanthine oxidase to produce superoxide radicals which then react with nitro-blue tetrazolium (NTB) to form a colored formazan dye. The amount of formazan generated was determined by the absorption at the wavelength of 550 nm. One unit of enzyme was defined as the amount of enzyme required at an inhibition rate of 50%. Enzyme specific activity was expressed in units per milligram protein.

#### 4.5.3. Determination of CAT Activity

The activity of CAT was assayed based on the method previously described [47]. Briefly, 0.1 mL of supernatant of tissues in hippocampus and cortex was added to 1.91 mL of 50 mmol/L phosphate buffer (pH 7.0). Then 1 mL freshly prepared 30 mmol/L H_2_O_2_ was added to start the reaction. The decrease in H_2_O_2_ content was determined by the absorption at the wavelength of 240 nm.

#### 4.5.4. Determination of GSH

The concentration of GSH was assayed according to a previous method [48]. In brief, 160 μL of supernatant of tissues in hippocampus and cortex was added to 2 mL of Ellman’s reagent (5, 5′-dithiobis [2-nitrobenzoic acid] 10 mM, NaHCO_3_ 15 mM). The mixture was incubated at room temperature for 5 min and the absorption was measured at the wavelength of 412 nm.

### 4.6. Enzyme-linked Immunosorbent Assay (ELISA)

The frozen brain cortex and hippocampal tissues were homogenized in ice-cold normal saline and centrifuged at 12,000 rpm at 4 °C for 5 min. The supernatants were then collected, and the total protein concentration was assayed using Micro BCA procedures (Beyotime Institute of Biotechnology, Shanghai, China). On the basis of the manufacturer’s instructions, enzyme-linked immunosorbent assay (ELISA) kits (Immuno-Biological Laboratories Co., Ltd., Japan) were used to quantify TNF-α and IL-6 in the supernatants.

### 4.7. Western Blotting

The frozen cerebral cortex and hippocampus tissues were homogenized in ice-cold extraction buffer (20 mM Tris-HCl buffer, pH 7.6, 150 mM NaCl, 2 mM EDTA·2Na, 50 mM sodium fluoride, 1 mM sodium vanadate, 1% Nonidet P-40, 1% sodium deoxycholate, 0.1% SDS, 1 mg/mL aprotinin, and 1 mg/mL leupeptin). The resultant homogenates were centrifuged at 10000× *g* for 10 min at 4 °C to obtain the final supernatants. Nuclear and cytoplasmic extracts for Western blot analysis were extracted using a nuclear/cytoplasmic isolation kit (Beyotime Institute of Biotechnology, Shanghai, China). Pierce BCA Protein Assay Kit (ibid.) was used to determine protein concentrations. Equal amounts of protein (20 μg) for each sample were separated by SDS–PAGE and transferred onto nitrocellulose membranes. And 5% skim milk powder in Tris-buffered saline containing 0.05% (v/v) Tween 20 (TBST) was used to block the membranes at 25 °C for 2 h, before incubation with the primary antibodies to NF-κB p65 (1:1000), p38 MAPK (1:1000), phospho- p38 MAPK (1:1000), SYN (1:1000), and PSD-95 (1:2000) and β-actin (1:1000) at 4 °C overnight. Then, the membranes were washed three times every 15 min with TBST and then incubated with the secondary horseradish peroxidase-linked anti-rabbit (1:1000) or anti-mouse (1:1000) antibodies (ZSGB-BIO, Beijing, China) at 37 °C for 1 hour. Bands were scanned, and the density was analyzed by the Quantity One analysis software (Bio-Rad Laboratories, Hercules, CA, USA). All quantitative analyses were performed based on our former researches [49].

### 4.8. Reverse Transcriptase-PCR (RT-PCR)

The assay was performed based on previous researches [44,48]. The total RNA was extracted using trizol reagent. A High Capacity RNA-to-cDNA kit was applied to synthesize cDNA. The sequences of the forward and reverse primers for SYN, PSD-95 and the housekeeping gene β-actin (Sangon Biotech Co. Ltd., Shanghai, China) are shown in Table 1. Electrophoresis on a 1% agarose gel was used to separate amplified products followed by photography for visualization under a UA trans-illuminator. In order to verify reproducibility, duplicate reaction was performed. The values obtained for the target gene expression were normalized to β-actin and quantified relative to the expression in the control samples. The products were analyzed with densitometry using the Quantity One 1-D analysis software (Bio-Rad, Hercules, CA, USA).

### 4.9. Histological Analysis

After the behavioral test, mice were immediately anesthetized with sodium pentobarbital (50 mg/g, i.p. injection) and then perfused with ice-cold normal saline followed by 4% paraformaldehyde via the left ventricle. The whole brain was removed and fixed in 4% paraformaldehyde, then in 15% cane sugar for 24 h, followed by dehydration in 30% cane sugar for 12 h. For histological analysis with Nissl’s staining, all specimens were frozen and cut into consecutive coronal sections (30 μm in thickness). The number of intact cells in the cerebral cortex and hippocampal CA1 subfield were counted by an investigator blinded to sample identity, and the average value from adjacent two sections was used for each animal. Data were represented as cells per mm^2^. The histological analysis was performed as previous research described [50].

### 4.10. Molecular Docking Studies

In order to investigate the possible binding modes of ATX with human p38 alpha and human p38 alpha with p38 inhibitor PH797804, a molecular docking study was carried out using the Sybyl v7.1 program package (Tripos International, St. Louis, MO). The three-dimensional structure of human p38 and p38 inhibitor PH797804 alpha were taken from the Protein Data Bank (PDB ID: 4l8m; http://www.rcsb.org/), hydrogen atoms were added to the crystallographic structures and all the water were removed subsequently. The energy of human p38 and p38 inhibitor PH797804 alpha were minimized, before ATX had been docked into the active site of the ATP pocket of p38 alpha.

### 4.11. Statistical Analysis

All values are expressed as the mean ± SEM and analyzed by SPSS v16.0 (SPSS, Inc., Chicago, IL, USA). Differences between the groups were assessed by the one-way ANOVA and the Turkey’s test. Significant differences were represented as * *p* < 0.05.

## Figures and Tables

**Table 1:** Sequences and annealing temperatures of the oligo primers used in this study.

Target mRNA Sequences	Primer Sequence	Annealing Tm (°C)
β-actin	5′ ATGGTCACGCACGATTTCCC 3′ 5′ GAGACCTTCAACACCCCAGC 3′	59
SYN	5′-TCTTCCTGCAGAACAAGTACC-3′ 5′-CCTTGCATGTGTTCCCTGTCTG-3′	200
PSD-95	5′- CCCAGACATCACAACCTCAT -3′ 5′- ACACCATTGACCGACAGGAT -3′	324
